# Microstructure of Sheared Entangled Solutions of Semiflexible Polymers

**DOI:** 10.3390/polym8100353

**Published:** 2016-09-28

**Authors:** Marc Lämmel, Evelin Jaschinski, Rudolf Merkel, Klaus Kroy

**Affiliations:** 1 Institut für theoretische Physik, Universität Leipzig, Postfach 100920, 04009 Leipzig, Germany; marc.laemmel@itp.uni-leipzig.de; 2 Institute of Complex Systems 7: Biomechanics, Forschungszentrum Jülich, 52425 Jülich, Germany; evelin.jaschinski@gmail.com

**Keywords:** shear alignment, F-actin, tube model

## Abstract

We study the influence of finite shear deformations on the microstructure and rheology of solutions of entangled semiflexible polymers theoretically and by numerical simulations and experiments with filamentous actin. Based on the tube model of semiflexible polymers, we predict that large finite shear deformations strongly affect the average tube width and curvature, thereby exciting considerable restoring stresses. In contrast, the associated shear alignment is moderate, with little impact on the average tube parameters, and thus expected to be long-lived and detectable after cessation of shear. Similarly, topologically preserved hairpin configurations are predicted to leave a long-lived fingerprint in the shape of the distributions of tube widths and curvatures. Our numerical and experimental data support the theory.

## 1. Introduction

Semiflexible polymers are fundamental structural and functional building blocks of biological matter. They are the main constituents of the dynamic cytoskeletal networks and extracellular matrices that maintain the cell’s mechanical stability and integrity. By controlling the mesoscale architecture of these scaffolds, cells regulate their response to mechanical load, and living organisms realize a wide range of mechanical properties and functions using only relatively few polymeric constituents [1,2]. Networks of semiflexible polymers are therefore at the core of many attempts to understand the rich mesoscopic and macroscopic mechanical response of biological matter in terms of its molecular machinery [3]. Over the last decades, great progress has been made by studying reduced in vitro model systems that share many macroscopically observed features of the far more complex natural systems [3,4,5,6]. Their mesoscale architecture is commonly characterized by a small set of mesoscopic parameters such as mesh size, polymer bundle thickness, and crosslinker concentration [7,8,9].

The classical rheological model for entangled solutions of flexible polymers is the so-called tube model [10,11]. It reduces the complex many-body problem to a simple mean-field picture featuring a test polymer in a long-lived tube-shaped confining cage, and thereby light-handedly accounts for some gross features of the linear rheology. Additional considerations are required to address the more intricate nonlinear rheology. For example, finite-chain stretching due to intermolecular friction [12] and tube contraction [13] were proposed to cause shear-stiffening and a rate-dependent stress overshoot during shear startup; (convective) constraint release [14] and similar concepts [15] were proposed to account for shear-softening.

One may expect similar ideas to apply to the much stiffer biopolymers that govern the mechanics of biological cells and tissues. Indeed, biopolymer solutions are generally reported to exhibit strain-softening and shear-thinning [16,17,18,19] under slow or stationary shearing, in accordance with predictions [20,21] based on a version of the tube model adapted to semiflexible polymers [22]. Again additional concepts, such as transient entropic filament stretching excited by some interchain friction due to transient filament crosslinking [1,23], were invoked to account for observations of transient shear-stiffening in response to large finite shear strains at higher rates [18,19,23,24,25]. Alternatively, fiber-lattice or “mikado” models for crosslinked networks relate the macroscopic stiffening not to the response of the individual constituents (modeled as linear elements) but to the collective behavior of a sparse sub-isostatic network [26]. An imposed strain is first taken up by so-called floppy modes [27,28], before the network becomes rigid at a critical strain [29,30]. Brownian-dynamics simulations [31] support the notion that stiffening at intermediate timescales (when the individual fibers have locally equilibrated) is due to slower collective modes.

Recent experiments combining bulk and microrheology measurements with confocal fluorescence microscopy and particle tracking techniques were able to record the conformation and orientation of individual filaments under shear [17,32,33,34,35], and found them to be very sensitive to the polymer concentration. In very dilute solutions, actin filaments perform a tumbling motion, switching between an advective and a diffusive phase, corresponding to strongly buckled (U-shaped) and stretched polymer conformations, respectively [34]. In the semi-dilute regime, tumbling is modified by filament–filament collisions, increasing both filament alignment [35] and bending [17], as evidenced by broad tails in the distribution of the local filament curvature. Such observations challenge mesoscopic theories and computer simulations [26,36,37,38,39] to extend the simple network models and also consider densification [33], ordering and alignment [17,40], and even lengthening [41] of fibers.

In the present paper, we address two major effects of shear onto the constituent polymers, namely alignment and bending, on the basis of the tube model of semiflexible polymers. In the following section, we derive theoretical predictions for the affine and non-affine shear alignment and the tube-width and curvature distributions in a sheared semiflexible polymer solution. They are then tested by dedicated computer simulations and experiments with semidilute F-actin solutions. Technical details are deferred to Section 4.

## 2. Results and Discussion

As pointed out in the introduction, shearing a semidilute polymer solution will generally deform and align the individual polymers and their long-lived tube-shaped confinement cages. In the following, we study these two effects separately. We first concentrate on the effect of an externally imposed nematic tube alignment on the local packing structure, quantified in terms of the tube width, in Section 2.1. We then ask how much alignment is actually caused by shearing. Following Morse [20] and Fernández et al. [21], we moreover estimate the tube deformation due to large finite shear strains by minimizing a model free energy and analyze the consequences for the tube-width and curvature distributions, in Section 2.2. Finally, we corroborate our key theoretical predictions by computer simulations and experiments with F-actin solutions, in Section 2.3 and Section 2.4.

### 2.1. Tube Alignment

In the absence of crosslinking molecules, the structure and mechanics of the transient polymer network that dominates the mechanical response of a semidilute biopolymer solution is governed by long-lived topological entanglements. They constrain the thermal motion of each constituent filament so that it remains effectively confined to a tube-like cage formed by surrounding filaments [42]. Shear is expected to cause some alignment of the polymers and their confining tubes, which is otherwise not entropically favorable below the nematic transition, and thereby to widen the tubes. In the following, we extend the so-called binary-collision approximation (BCA) developed in the classical tube model of semiflexible polymers [43] to quantify the effect theoretically.

As usual, the polymer solution is characterized by its chain length concentration *ρ*, the persistence length $L_{p}$ of the constituting polymers, and the nematic order parameter

(1) $$S=\left(3/2\right)\int \;\;du\;{\left(u\;\cdot \;d\right)}^{2}f_{S}\left(u\right)-1/2\;.$$

Here, $f_{S}\left(u\right)$ denotes the distribution of the normalized tangent vector u of the filaments or tubes, respectively, and d their mean direction. Extending standard procedures [43,44], as detailed in Section 4.3, we arrive at the following BCA predictions for the mean tube radius and entanglement length for a solution of prealigned polymers:

(2) $$\begin{array}{cc} \bar{R}\left(S\right) & ={\left[4\alpha_{0}I\left(S\right)\rho \right]}^{-3/5}L_{p}^{-1/5}∼\bar{R}\left(0\right)\left(1+3S^{2}/8\right)\;,\\ \bar{L_{e}}\left(S\right) & ={\left[\alpha_{0}I\left(S\right)\rho /8\right]}^{-2/5}L_{p}^{1/5}∼\bar{L_{e}}\left(0\right)\left(1+S^{2}/4\right)\;. \end{array}$$

Here, I(S) is the normalized packing entropy of the solution, which we estimate by Onsager’s rigid-rod prediction [45] to derive the final asymptotic expressions, valid for weak alignment (small *S*). A numerical solution of the full theory corroborates what the asymptotic expressions suggest, namely that strong alignment (S→1) is required to induce any sizeable tube widening. This is in line with the prediction $\bar{R}\left(S\right)/\bar{R}\left(0\right)\propto I{\left(S\right)}^{-1/2}$ for rigid rod solutions, as derived by Doi and Edwards from geometrical arguments based on pair collisions [11] and by Sussman and Schweizer [46] building on the binary-collision approach to rigid-rod solutions by Szamel [47]. The caging of rigid rods and the entropic repulsion and attraction induced by the conformational fluctuations of semiflexible polymers, encoded in Equation (2), thus yield quantitatively similar predictions. Our quantitative result is compared to our experiments and simulations in Section 2.3, below.

In References [44,48], the BCA scheme was generalized to the so-called segment-fluid approximation that gives access to the tube fluctuations as encoded in the distributions P(R) and $P(L_{e})$ of both the tube width and the entanglement length. The predictions were found to be in good agreement with experimental data obtained from partially fluorescently labeled F-actin solutions, allowing for a decent global fit for various actin concentrations. Within our extended version of the BCA with preferential filament alignment (detailed in Section 4), the distribution functions for the reduced variables $r\equiv R/\bar{R}$ and $l_{e}\equiv L_{e}/{\bar{L}}_{e}$ take the form of universal scaling functions that are not only independent of concentration but also of the nematic order parameter *S*. Moreover, the distribution of the entanglement length, which is the characteristic correlation length (of the local tangent orientations, tube widths, curvatures, etc.) along the tube backbone, is predicted to be strongly peaked around the its mean: $\bar{l_{e}^{2}}-1\approx 0.01$. As a consequence, our above discussion of the effect of alignment onto the solution rheology on the level of the mean values $\bar{R}$ and $\bar{L_{e}}$ should suffice.

To get a rough idea, how much alignment is actually caused by shearing an initially isotropic solution, we estimate the alignment of short, relatively straight tube segments from the affine response of a solution of rigid phantom rods [45],

(3) S(γ)∼3γ/10(S<1).

**Figure 1:**
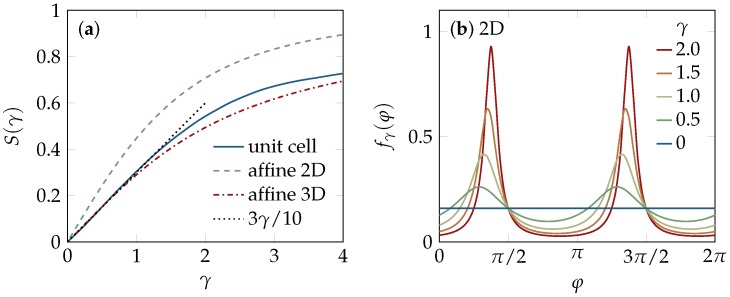
Shear alignment of tube segments. (**a**) Strain-dependence of the nematic order parameter S(γ): affine scaling, as obtained for two- and three-dimensional solutions of phantom rods (Section 4.4), and the numerical estimate from the unit-cell model [21] (see Section 4.5). Up to strains of order one, the results are well captured by the linear asymptotic scaling of Equation (3), while S(γ) flattens out for larger strains, implying that perfect shear alignment is hard to achieve, even if quite substantial strains are imposed; (**b**) The angular distribution of the two-dimensional phantom-rod solution, according to Equation (19). With increasing strain the bimodal structure becomes more pronounced.

The linear increase with applied strain is dictated by symmetry [38] and thus more general than its derivation, which is detailed in Section 4.4, where we additionally derive the corresponding angular distribution of the tube-segment directions. Beyond the linear asymptotic regime, we find a considerable flattening of S(γ) at about γ≳3, as illustrated in Figure 1. Shear alignment beyond S≈0.7 is thus very hard to achieve. Now, using the result for S(γ) in Equation (2), we obtain the following prediction for the strain-dependence of the tube radius and entanglement length due to shear alignment,

(4) $$\begin{array}{cc} \bar{R}\left(\gamma \right)/\bar{R}\left(0\right) & ∼1+0.034\gamma^{2}\;,\\ \bar{L_{e}}\left(\gamma \right)/\bar{L_{e}}\left(0\right) & ∼1+0.023\gamma^{2}\;. \end{array}$$

The small numerical coefficients show that both quantities are weakly affected even by quite substantial shearing, as far as shear alignment is considered. This is indeed also borne out by our computer simulations and experiments discussed in Section 2.3, below. As a consequence, also the restoring forces associated with shear alignment should be weak. For this reason we expect it to persist long after a large finite shear deformation has been applied. However, shearing affects the packing structure of the polymer solution not only through shear alignment, but also through (non-affine) tube deformations, for which more sizeable rheological consequences were indeed predicted by Morse [20] and Fernández et al. [21]. These are analyzed in the next paragraph.

### 2.2. Tube Deformation

The extended BCA theory used in the above calculation is an effective two-body theory and thus blind to the complicated many-body effects involved in shearing. The unit-cell approach by Fernández et al. [21] considers a test polymer together with two collision partners located on opposite sides, instead (see Section 4.5), and can thereby capture some geometric aspects inaccessible to the BCA. In particular, it predicts non-affine deformations of the microstructure, because only the tube–tube collision points (or, alternatively, the centers of the confining tubes) are slaved to the affine deformation field, whereas the backbone contour of the considered test tube relaxes to a (non-affine) conformation that minimizes the unit-cell free energy. As a consequence, the strain-dependent order parameter S(γ) may generally be expected to differ from the affine estimate in Equation (3). But we find good agreement between both predictions for moderate strains γ<1, and even for the saturation at large strains (beyond γ≈3), as detailed in Section 4.5 and illustrated in Figure 1. The non-affine contributions merely slightly enhance the alignment at intermediate strains. Altogether, the unit-cell model thus confirms the above phantom-rod prediction that shear alignment is effectively bound to remain relatively moderate (S≲0.7), even up to quite substantial strains of several hundred percent.

Importantly, though, the unit cell model predicts sizable non-affine deformations of the local packing structure, beyond the pure shear-alignment effect. For example, we find for the strain dependence of the tube radius:

(5) $$\bar{R}\left(\gamma \right)/\bar{R}\left(0\right)∼1+b_{R}\gamma^{2}\;.$$

Here and in the following, we use the script typeface to discriminate the quantities calculated from the unit-cell model from the above BCA estimates. An important difference between them, even if no shear is applied, is that the BCA conventionally considers a straight tube, whereas the unit-cell model allows tubes to bend if this lowers the total equilibrium free energy, which balances contributions from confinement *and* bending. In Section 4, we find that the average tube width thereby grows by a factor $\bar{R}\left(0\right)/\bar{R}\approx 1.4$ in the quiescent solution, and even further, with the coefficient $b_{R}\approx 0.14$, upon shearing. The comparison of Equations (4) and (5) thus suggests that the nonlinear shear-softening of entangled polymer solutions and the associated rheological stresses are predominantly caused by non-affine tube deformations with only minor contributions from shear alignment. In view of the above-established flattening of S(γ) at large strains, this statement is likely to hold beyond the range of validity of the asymptotic result in Equation (5).

Similarly, we can use the unit-cell model to quantify how shearing affects tube bending. For the mean curvature of the tube backbone we find for small deformations:

(6) $$\bar{C}\left(\gamma \right)/\bar{C}\left(0\right)∼1+b_{C}\gamma^{2}\;.$$

with $\bar{C}\left(0\right)\approx 1.4{\left(L_{p}\bar{L_{e}}/2\right)}^{-1/2}$, where ${\left(L_{p}\bar{L_{e}}/2\right)}^{-1/2}$ is the mean curvature of a wormlike chain confined to a straight tube segment of length $\bar{L_{e}}/2$, the coefficient $b_{C}\approx 0.037$ is obtained. Its small positive value indicates that, on average and for moderate strains, the effect of filament buckling slightly exceeds that of filament stretching.

The average curvature of the tube can also be quantified by a tube persistence length $l_{t}$, conveniently inferred from the Odijk relation ${\bar{L_{e}}}^{3}=4^{3}{\bar{R}}^{2}l_{t}$ between entanglement length and tube width [49]. For a straight tube, $l_{t}$ is equal to the bare intrinsic persistence length $L_{p}$ of the enclosed test polymer. However, as already pointed out above, the unit cell model predicts a substantial renormalization, even without shear, because it allows the tube to bend spontaneously to minimize the unit-cell free energy, in qualitative accord with the persistence-length renormalization due to molecular crowding found in recent model simulations [50]. Quantitatively, we find $l_{t}\left(0\right)={\left[\bar{R}/\bar{R}\left(0\right)\right]}^{2}L_{p}\approx 0.56L_{p}$, in line with our above finding $C\left(0\right)\approx 1.4{\left(L_{p}\bar{L_{e}}/2\right)}^{-1/2}$, which can thus be rewritten as $C\left(0\right)\approx {\left(l_{t}\left(0\right)\bar{L_{e}}/2\right)}^{-1/2}$. Our own simulations cannot reach high enough densities to make this effect discernible. If we extend the Odijk relation to the case of a sheared solution, namely $\bar{L_{e}}{\left(\gamma \right)}^{2}=4^{3}\bar{R}{\left(\gamma \right)}^{2}l_{t}\left(\gamma \right)$, and replace $\bar{L_{e}}\left(\gamma \right)$ by its equilibrium value $\bar{L_{e}}$ (which is a good approximation for moderate strains), we find for the renormalization of the tube persistence length under shear

(7) $$l_{t}\left(\gamma \right)/L_{p}∼{\left[\bar{R}/\bar{R}\left(\gamma \right)\right]}^{2}={\left[\bar{R}/\bar{R}\left(0\right)\right]}^{2}{\left(1+b_{R}\gamma^{2}\right)}^{-2}\;.$$

So, some polymers stretch and others buckle upon shearing, but, overall, buckling wins and the average tube persistence length decreases, in line with the increasing curvature, found above.

A more comprehensive characterization than by mean values is possible by statistical distribution functions. In contrast to the marginal effects that we obtained from tube alignment, above, we now find the distributions to be quite sensitive to the non-affine shear deformations predicted by the unit-cell model. Rephrasing the result in terms of the reduced tube-radius distribution $p_{\gamma }\left(r\right)=\bar{R}\left(\gamma \right)P_{\gamma }\left[r\bar{R}\left(\gamma \right)\right]$ yields a master curve onto which the appropriately normalized experimental data should collapse, independently of the actin concentration. For the curvature distribution, we apply the same procedure to arrive at a reduced curvature distribution $p_{\gamma }\left(c\right)$.

**Figure 2:**
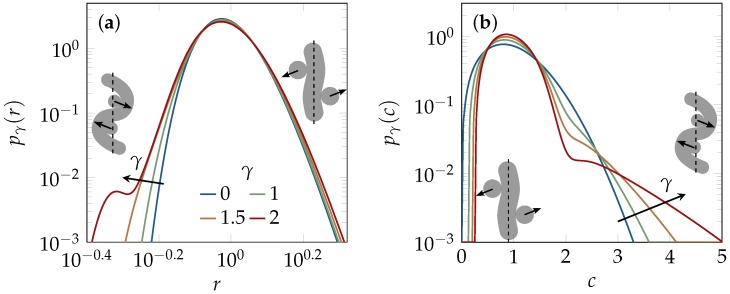
How shear affects packing structure in the unit-cell model [21]. In contrast to the most probable tube conformations, rare hairpin configurations are buckled and pulled tighter by increasing shear (as sketched in the insets). They are responsible for the tails emerging upon increasing strain *γ* in the concentration-independent master curves of the reduced probability distribution functions (**a**) $p_{\gamma }\left(r\right)\equiv \bar{R}\left(\gamma \right)P\left[r\bar{R}\left(\gamma \right)\right]$ for the tube width and (**b**) $p_{\gamma }\left(c\right)\equiv \bar{C}\left(\gamma \right)P_{\gamma }\left[c\bar{C}\left(\gamma \right)\right]$ for the tube curvature.

The predicted influence of shear on the master curves corresponding to $p_{\gamma }\left(r\right)$ and $p_{\gamma }\left(c\right)$ is illustrated in Figure 2. It reveals that $p_{\gamma }\left(r\right)$ develops broad tails at small arguments as the strain *γ* increases, whereas $p_{\gamma }\left(c\right)$ becomes more sharply peaked around its average (normalized to 1) and develops a tail at large arguments. The emergence of the tails can be traced back to so-called hairpin conformations (thermodynamically suppressed strongly contorted unit cell configurations [20,21]), as schematically sketched as insets in Figure 2. They are pulled tight under shearing, which accounts for the increasingly bimodal structure developing for large strains *γ* in both distributions, but is found to have only negligible impact onto the mean tube parameters.

### 2.3. Experiments and Simulations

The details of our simulations and experiments can be found in Section 4. Briefly, the simulations use a hybrid Monte-Carlo/Brownian-Dynamics algorithm, developed by Ramanathan and Morse [51,52,53], who kindly provided us with the source code of their program, to sample over topologically allowed states of a solution of wormlike chains. In this algorithm a sequence of Monte-Carlo steps, which respect the mutual uncrossability of colliding chains, is drawn from the stochastic dynamics of each chain, as obtained by solving a corresponding Langevin equation. The polymers were given a preferential orientation at initialization, i.e., before the uncrossability constraints and Brownian motion were switched on.

**Figure 3:**
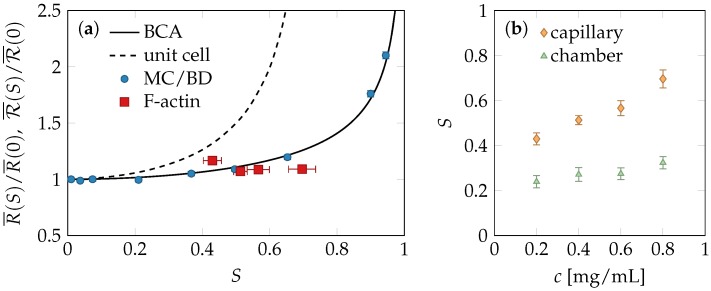
Nematic alignment and tube deformation. (**a**) Dependence of the mean tube radius $\bar{R}$ on the nematic order as predicted by the binary-collision approximation (BCA) calculation, Equation (2), and the unit-cell model. The four experimental data points correspond to four different F-actin concentrations *c*. Our Monte-Carlo/Brownian-Dynamics (MC/BD) simulations of pre-aligned polymer solutions and F-actin experiments show no sign of the strong strain-induced tube dilation predicted by the unit-cell model but agree with the BCA predictions for moderately pre-aligned tubes, corresponding to shear alignment by a strain of about γ=1.5⋯2.5. We interpret this as an indication that the average tube deformations had mostly relaxed between the cessation of shear and the start of the measurements, while the inflicted shear alignment was largely conserved. Note that the statistical errors of the tube size is very small (≈ 1%) for both the experiments and the simulations; (**b**) Polymer solutions were prepared in two different sample geometries for each *c*, a narrow capillary and a wider micro chamber, to get strongly sheared networks and weakly sheared reference samples, yielding values for $\bar{R}\left(S\right)$ and $\bar{R}\left(S\approx 0\right)$, respectively. Their ratio is shown in panel (**a**) against the values for *S* in the capillary.

In the experiments, the thermal motion of a fluorescently labeled actin filament in the meshwork of unlabeled neighbor filaments is tracked over a fixed time span, long enough to identify the shape of the confinement tube. Two different setups were used to prepare the samples: a large micro chamber and a narrow capillary yielding almost isotropic and nematically ordered solutions, respectively. Varying the polymer concentration, this approach provides the dependence of the tube width on the alignment strength *S*, because the flow-induced ordering depends on the concentration—denser solutions yielding stronger alignment, see Figure 3b. The measured relation between the average tube width and alignment displayed in Figure 3a is consistent with the simulations and the BCA-prediction.

**Figure 4:**
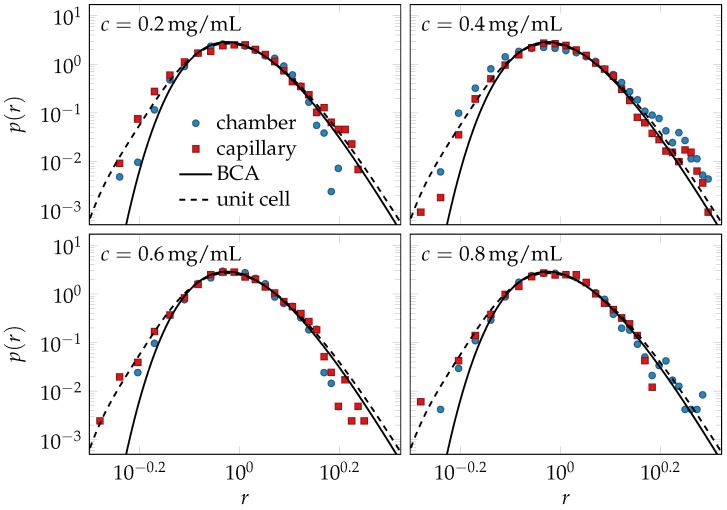
Reduced tube-width distribution: differently prepared F-actin experiments collapse onto a single master curve $p\left(r\right)=\bar{R}P\left(r\bar{R}\right)$, independent of both concentration and the degree of nematic order of the solution. The scaling and the shape of the equilibrium master curve, Equation (12), are predicted by the tube model, evaluated in the binary-collision approximation (BCA). Its deformation due to shearing is estimated using the unit-cell model. Small deviations between the data and the equilibrium theory are consistent with the predicted effect of a remnant strain γ=1.5 (dashed lines) and interpreted as indicative of long-lived deformations of rare hairpin configurations.

Beyond the mean tube width, we also measured the distribution of tube widths. As shown in Figure 4, the rescaled data for all concentrations and *S*-values fall on a master curve, as predicted by the extended BCA.

### 2.4. Comparison of Theory and Data

Our theoretical, experimental, and numerical findings all suggest that moderate tube-segment alignment only weakly influences the tube size and its distribution and therefore excite only weak restoring stresses. Curiously, all our experimental data seem to fall into regime of moderate alignment, up to S≈0.5 where the tube radius is almost independent of *S*, cf. Figure 3. The experimental data support the predictions obtained from both the simulations of the prealigned fiber solutions and the affine phantom model and BCA prediction: the detected alignment is compatible with an average tube width equal to its equilibrium value.

The shear strain *γ* imposed on the actin solution cannot directly be controlled, in our setup, but from the recorded filling speeds of the capillary, it should be similar for all analyzed actin concentrations. By the time of the measurement, the solution was no longer actively sheared, only the final shear strain was maintained. According to Equation (3), the observed weak alignment corresponding to nematic order of strength S≈0.4⋯0.7 is consistent with remnant shear strains γ≈1.5⋯2.5 and reflects the predicted difficulty to achieve any stronger shear alignment with such strains. At the same time, the measured tube-radius data show no sign of the sizeable increase of the mean tube radius $\bar{R}\left(S\right)$ predicted by the unit-cell model as a consequence of tube deformations. A plausible explanation could be that the tube deformations had already been undone by the associated restoring stresses at the time of the measurements, whereas the negligible restoring stresses associated with the experimentally observed moderate tube alignment allowed the latter to persist. Indeed, having no discernible effect on the tube conformations, these stresses should not appreciably exceed the thermal energy per tube volume.

**Figure 5:**
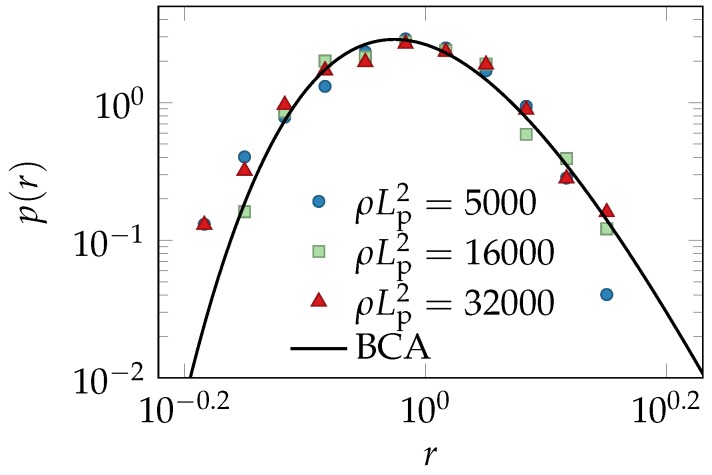
Reduced tube-width distribution obtained from the hybrid Monte-Carlo/Brownian-dynamics computer simulations [51,52,53]. As expected from the BCA prediction, data for various polymer length concentrations *ρ* collapse onto a single master curve.

Another effect on the packing structure that should arguably be long-lived and experimentally detectable is the change in the tube-width and curvature distributions caused by the shearing of hairpins (Figure 2). Hairpin configurations are topologically prevented from relaxing into more typical configurations without first disentangling from their tubes. They are preserved and even stabilized upon shearing, and their effects onto the shapes of the tube-width and curvature distributions are independent of the average values of the tube width and curvature. Hence, they also should relax on a very slow time scale, and their deformation by shear and its characteristic fingerprint in the distributions in Figure 2 (relative enhancement of the fraction of small tube radii and large tube curvatures) should be largely preserved after cessation of shear, when the average tube width and curvature have already relaxed. Indeed, as demonstrated in Figure 4, the frequency of small tube widths is found to be increased compared to the prediction of the equilibrium model (solid lines). Excellent agreement of theory and data for the tube-width distribution is obtained by choosing a plausible value for the remnant strain γ=1.5, consistent with the observed tube alignment in Figure 3, according to Equation (3). Despite this very favorable agreement, some issues remain to be resolved. Our computer simulations seem to indicate a tendency of the BCA to systematically underestimate the fraction of narrow tubes, even in equilibrium solutions (Figure 5 of the methods section). A thorough investigation of this issue is currently hampered by computational limitations and experimental difficulties. The measured tube width distributions have a tendency to weakly broaden within the observable finite-time windows, presumably because the ideal limit of strong entanglement is difficult to achieve in practice (especially in computer simulations).

## 3. Conclusions

The mechanical properties of entangled solutions of semiflexible polymers depend crucially on the response of the mesoscopic architecture to external perturbations. We have analyzed the impact of two such perturbations: an imposed affine nematic odering and a proper shear deformation that induces a similar degree of nematic alignment but also additional, non-affine strains. By measuring the tube-shaped cages of labeled test polymers after cessation of shear, we found that initially isotropic solutions developed moderate nematic order by shear alignment, which persisted after the shearing had stopped. Besides this shear-alignment, which is comparable to what one would expect from a purely affine model, shearing was predicted to cause non-affine local tube deformations (Figure 3) and leave a characteristic fingerprint in the tube-width and backbone-curvature distributions (Figure 2). We estimated both effects using the unit-cell approach by Fernández [21]. We could not detect the expected average tube deformations, experimentally, presumably because they had been driven back by the associated restoring forces, at the time of measurement. However, our data for the reduced tube-width distribution could well be fitted by the unit-cell model, assuming a finite remnant strain consistent with the observed tube alignment (Figure 4). The theory identifies a small fraction of topologically protected hairpins as the main source of the observed deviations from the equilibrium distributions. Similarly, literature data for the microstructure of F-actin solutions [17,54] seem consistent with a sizeable influence of shear on the curvature distribution $p_{\gamma }\left(c\right)$, although the very large strains imposed in Reference [17] prohibit a direct comparison.

In summary, our experimental and numerical data can be reconciled with the predictions of the unit-cell model if one accepts that the predicted average tube dilation and tube buckling upon shearing is energetically costly and relaxes quickly, so that it is not detectable after cessation of shear, whereas moderate tube alignment and hairpin deformations induce no sizeable (global) stresses and are therefore longer-lived, hence detectable. With this interpretation, our comparison of theory and experiment yielded consistent results but calls for further investigations. It would be particularly interesting to test the predicted faster relaxation of the average tube width and curvature as opposed to the shear alignment and hairpin effects with a higher time resolution as possible in our setup. A careful analysis of the evolution of curvature distributions upon application of finite large strains would also be very desirable.

## 4. Materials and Methods

### 4.1. Experiments

Actin was isolated from rabbit skeletal muscle, purified, and polymerized following standard procedures [48] to gain F-actin solutions of polymer concentrations *c* in the range from 0.2 to 0.8 mg/mL. These values correspond to dimensionless polymer length concentrations $\rho L_{p}\approx 2300$ to 9200, based on the typical value $L_{p}=17\;\mu m$ of the persistence length and the estimate $\rho /c\approx 40\;\mu m^{-2}/\left(\text{mg}/\text{mL}\right)$ obtained from the molecular structure of the actin filaments [43].

The filaments were labeled with TRITC-Phalloidin (Sigma Aldrich, Taufkirchen, Germany) and mixed gently with unlabeled filaments at a ratio of 1:1000. We used two different sample geometries for each concentration, a narrow capillary (0.1 mm×2 mm×50 mm, CM Scientific Ltd., West Yorkshire, UK) and a large chamber (8 mm×8 mm×5 mm, Lab-Tek Chambers, Nalge Nunc International, New York, NY, USA), yielding nematically ordered and almost isotropic polymer networks, respectively. Two-dimensional confocal microscope (LSM510, Carl Zeiss, Jena, Germany; objective C-Apochromat 63x/1.2 W korr; 543 nm laser and long pass filter 560 nm) images of a fluorescently labeled filament were recorded every second during a time span of 150 s, superimposed, and analyzed to measure the tube width, i.e., the space explored by the fluctuating polymer, similar to previous studies [48]. Due to the large number of data points that we collected, the statistical errors for the mean tube width, as obtained from a standard Jackknife method, are on the order of 1%. Filament orientations (i.e., order parameters) were obtained from three-dimensional stacks of images with voxel sizes chosen according to optical resolution and Nyquist’s sampling theorem.

### 4.2. Simulations

We use a hybrid Monte-Carlo/Brownian-dynamics algorithm proposed by Ramanathan and Morse [51,52,53] to simulate networks of entangled wormlike chains that have zero thickness but can not cross each other. The Brownian dynamics of each bead-rod polymer in the solution is computed by numerically integrating the corresponding Langevin equations. Each time step a trial move is computed for one randomly chosen polymer and steric interactions between filaments are implemented by rejecting the trial move if it would lead to a cut through a neighbor filament.

To mimic the shear alignment observed in the experiments we implement nematic order in the simulations by an external field -hcos(γ) that favors nematic alignment with an external director. The field acts during the initialization phase when the polymers are generated and placed in the simulation box in their free equilibrium states. After the system is initialized, the field is turned off and the system evolves thermally— similar to what happens in experiments right after preparation. At the end of each simulation run, we measure the order parameter

(8) $$S=\frac{1}{2N_{\mathrm{rod}}}\sum_{i=1}^{N_{\mathrm{rod}}}\left(3\cos^{2}\theta_{i}-1\right)$$

of the network by averaging the orientations of the $N_{\mathrm{rod}}$ polymer segments. The direction of each polymer segment is characterized by the angle $\theta_{i}$ between a rod that connects two neighboring monomer beads and the externally imposed director. Following Reference [52], we determine the time-dependent tube radius R(t) from the reptation-corrected MSD,

(9) $$R{\left(t\right)}^{2}=\frac{1}{N(T-t)}\sum_{i=1}^{N}\int_{0}^{T-t}\;\;\;\;\;\;\;\;d\tilde{t}\;d_{i}{\left(\tilde{t},\tilde{t}+t\right)}^{2}\;,$$

where *N* is the number of molecules, *T* the total simulation time, and $d_{i}\left(\tilde{t},\tilde{t}+t\right)$ gives the closest approach between the chain’s middle bead at time $\tilde{t}+t$ and the contour of this chain at time $\tilde{t}$. The *S*-dependent equilibrium mean tube radius $\bar{R}\left(S\right)$ is then obtained assuming that $R\left(t\right)=\bar{R}\left(S\right)f\left(t/\tau_{e}\right)$ can be written in terms of a universal scaling function *f* [51], where the so-called entanglement time $\tau_{e}\propto \bar{R}{\left(S\right)}^{8/3}$ itself depends on the mean tube width.

The data shown in Figure 4 were obtained for solutions that contained 1296 chains of length $L=L_{p}$ in a cubic simulation box of edge length 1.2L, yielding a dimensionless polymer length concentration of $\rho L_{p}^{2}=750$ and an entanglement length on the order of $L_{e}\approx 0.2L_{p}$ according to Equation (11). The simulation time was set to one half the rotational diffusion time of a straight rod of length *L*, which corresponds to about $40\tau_{e}$ for the used polymer concentration. The measured order parameter *S* was found to remain almost constant during the whole simulation time.

Figure 5 compares the reduced distribution of the tube widths obtained from isotropic solutions of various concentrations with the BCA prediction.

### 4.3. Binary Collision Approximation (BCA)

We tackle the complicated many-body problem of an entangled network of semiflexible polymers using the binary collision approximation (BCA) [43], which can easily be extended to nematically ordered networks. A representative test polymer is modeled as a wormlike chain (WLC) of persistence length $L_{p}$. Its collisions with other polymers in the solution are accounted for as far as these can be represented by independent pair interactions. Collective many-body effects are summarily included on a mean-field level by confining all polymers to cylindrical harmonic cages by adding a term $\bar{\phi }r_{⊥}^{2}/2$ to the WLC-Hamiltonian. Balancing the bending and confinement free energy contributions, the tube radius, defined as the average transverse displacement ${\bar{R}}^{2}\equiv {\left(2L\right)}^{-1}\int_{0}^{L}dsr_{⊥}^{2}\left(s\right)$ of the test chain, and the entanglement length $\bar{L_{e}}\equiv \phi^{-1}{\bar{R}}^{\;-2}$, which characterizes the mean distance between tube–tube collisions along a tube backbone, follow from a straightforward calculation as

(10) $$\bar{R}=2^{-3/4}L_{p}^{-1/8}{\bar{\phi }}^{\;-3/8}\;\mathrm{and}\;\bar{L_{e}}=2^{3/2}L_{p}^{1/4}{\bar{\phi }}^{\;-1/4}\;,$$

respectively. It was proposed by Morse [43] that the mean tube strength $\bar{\phi }$ can be determined self-consistently within the BCA as an average over all possible polymer–polymer configurations. The strategy is to describe the steric interaction of two colliding chains in the solution as a function of the size of the tube each chain is confined to. The calculation is eventually closed by identifying the average of the so obtained pair interaction as the mean-field tube potential. More precisely, the collision geometry of the two polymer segments that fluctuate around their straight primitive paths is described in terms of their relative orientation and separation *x* (i.e., their closest approach). The strength $\phi_{\pm }\left(x\right)$ of the harmonic confinement potential is obtained as the second-order Taylor coefficient of the potential of mean force $F_{\pm }\left(x\right)$ (“BCA potential”), $\phi_{\pm }\left(x\right)\propto \partial_{x}^{2}F_{\pm }\left(x\right)$. The latter is of pure entropic origin and given as the negative logarithm of the partition sum of a pair of polymers, each dressed by its own tube, in either a topologically open (subscript “+”) or entangled (“−”) configuration. (See also the sketch in Figure 2 of Reference [44].) This differentiation is necessary, because the number of states for two bendable polymers is not completely determined by the positions and orientations of their primitive paths, as it would be the case for straight rigid rods [48]. From the average over all colliding segment pairs one obtains the relation $\bar{\phi }=\alpha_{0}\rho /\bar{R}$ that links $\bar{\phi }$ to the polymer contour-length concentration *ρ* and the mean tube radius $\bar{R}$. Together with Equation (10), this procedure yields the self-consistent BCA solutions

(11) $$\bar{R}={\left(4\alpha_{0}\rho \right)}^{-3/5}L_{p}^{-1/5}\;\mathrm{and}\;\bar{L_{e}}={\left(\alpha_{0}\rho /8\right)}^{-2/5}L_{p}^{1/5}$$

of the mean tube radius and the entanglement length, respectively, with a numerical coefficient $\alpha_{0}\approx 0.50$ [44].

In Reference [44,48], the above theory for an average tube was generalized to the so-called segment-fluid approximation that gives access to the distribution P(ϕ) of tube strengths, which can vary along the test chain. Its predicted statistics of tube-radius fluctuations was found to be in good agreement with experimental data, allowing for a decent global fit for various actin concentrations. The central idea behind the segment-fluid model is to introduce a canonical ensemble of N+1 independent entanglement segments of length *L*, each dressed by an individual tube associated with its own value of ϕ. The segment-averaged mean field $\bar{\phi }$ felt by the test polymer as a whole, is thus obtained as an average over the *N* collision partners. In general, any higher order moment $\bar{\phi^{k}}$ can be computed similarly, to estimate the complete tube-strength distribution P(ϕ). Glaser et al. [44,48] showed that the distribution P(ϕ) can be approximated to very good accuracy by a Gamma distribution with mean $\bar{\phi }=\alpha_{0}\rho /\bar{R}$ and variance $\bar{\phi^{2}}-{\bar{\phi }}^{2}=\beta_{0}\rho /\left(L{\bar{R}}^{3}\right)$, where $\beta_{0}\approx 0.094$. Within the tube-segment approach, the relation between network-averaged tube width and tube strength, given by Equation (10), is replaced by a similar relation for the fluctuating quantities *R* and ϕ, which allows to convert P(ϕ) directly into the tube-width distribution P(R). Rescaling the tube radius by its mean, a concentration-independent scaling form of the distribution [44]

(12) $$p\left(r\right)=\bar{R}P\left(r\bar{R}\right)\propto r^{-19.1}e^{-6.11r^{-8/3}}$$

ensues. Here, the magnitude $\bar{r^{2}}=\bar{R^{2}}/{\bar{R}}^{2}$ of the fluctuations is completely determined by the value of the combination $\left(L/L_{e}\right)\alpha_{0}/\beta_{0}$, and the scaled length $L/L_{e}\approx 1.3$ of the tube segment is obtained by comparing $\bar{r^{2}}$ with a corresponding fluctuation-response estimate, which is derived for a polymer exposed to an external force that is self-consistently identified with the confinement force. The latter calculation reveals that $L/L_{e}$ itself depends only on the ratio $\alpha_{0}/\beta_{0}$. Following the same lines that lead to the tube-width distribution, we can use the local version of Equation (10) to derive the distribution $P(L_{e})$ of the entanglement length from P(ϕ). Again, one obtains that the reduced distribution $p\left(l_{e}\right)=\bar{L_{e}}P\left(l_{e}\bar{L_{e}}\right)$ depends only on the value of $\left(L/L_{e}\right)\alpha_{0}/\beta_{0}$ and takes the universal form

(13) $$p\left(l_{e}\right)\propto l_{e}^{-28.0}e^{-6.14l_{e}^{-4}}\;.$$

We now extend the BCA to nematically ordered polymer solutions, characterized by the standard order parameter as defined in Equation (1). Repeating the calculations outlined in Reference [44] with this generalized orientational segment distribution, we find that the mean $\bar{\phi }$ and the variance $\bar{\phi^{2}}-{\bar{\phi }}^{2}$ of the tube strength take the same form as their isotropic-solution analogs, given in the text above Equation (12), but now with the functions

(14) $$\alpha \left(S\right)=\alpha_{0}I\left(S\right)\;\mathrm{and}\;\beta \left(S\right)=\beta_{0}I\left(S\right)\;,$$

that replace the numerical coefficients $\alpha_{0}$ and $\beta_{0}$, respectively, which yields the expressions for the mean tube radius and the mean entanglement length given in Equation (2). Here,

(15) $$I\left(S\right)=\left(4/\pi \right)\int \;\;du_{1}du_{2}\;f_{S}\left(u_{1}\right)f_{S}\left(u_{2}\right)\left|u_{1}\times u_{2}\right|$$

denotes the normalized packing entropy of the solution. For low nematic order, S≪1, the distribution $f_{S}\left(u\right)$ can be expanded up to linear order in *S*, for which the asymptotic proportionality $I\left(S\right)-1\propto S^{2}+O\left(S^{3}\right)$ follows from the normalization of $f_{S}\left(u\right)$ and the definition of *S*. It reveals that I(S), and thus $\bar{R}\left(S\right)$, varies only weakly with *S*, as long as the solution is not too strongly ordered. To make these dependencies more quantitative, we have to specify the distribution function $f_{S}\left(u\right)$. Since we expect its exact functional form not to be crucial, we choose Onsager’s trial function $f_{a}\left(\theta \right)=a\cosh\left(a\cos\theta \right)/\left(4\pi \sinh a\right)$ that was originally applied to solutions of rigid rods [45] and covers the wanted generic features of the distributions. Here, *a* is related to the order parameter via $S=1-3a^{-1}\coth a+3a^{-2}$, and $\theta =\cos^{-1}\left(u\;\cdot \;d\right)$ is the angle between the direction of the tube segment and the nematic axis. As shown by Onsager, this trial function yields $I\left(S\right)=2\;I_{2}\left(2a\right)/\sinh^{2}a$, where $I_{2}$ denotes the modified Bessel function of the first kind. Replacing $\alpha_{0}$ in Equation (11) by its order-dependent extension α(S), Equation (14), we obtain Equation (2) for the mean tube radius and entanglement length of the nematically ordered system, where the weak-order asymptotics in Equation (2) follow from $S∼a^{2}/15$ together with $I\left(S\right)∼1-a^{4}/360$.

### 4.4. Affine Strain Alignment

To estimate the tube-segment orientation in a sheared solution, we consider a solution of straight inflexible (phantom) rods that follow an externally applied shear strain *γ* affinely. We describe the latter in terms of the deformation matrix $\Lambda_{\gamma }$, so that the initial distribution $f_{0}\left(u\right)$ of the rod orientations u is changed to

(16) $$f_{\gamma }\left(u\right)={\left|\frac{du_{\gamma }}{du}\right|}^{-1}f_{a}\left[u_{\gamma }^{-1}\left(u\right)\right]\;,\;\mathrm{with}\;u_{\gamma }\left(u\right)=\frac{\Lambda u}{\left|\Lambda u\right|}\;,$$

where $u_{\gamma }:S^{d-1}\to S^{d-1}$, as well as its inverse $u_{\gamma }^{-1}\left(u\right)=\Lambda^{-1}u/\left|\Lambda^{-1}u\right|$, is an automorphism of the sphere of dimension d-1. Since the expressions for d=3 become quite lengthy, we first start with d=2, which already illustrates how shearing induces nematic ordering.

#### 4.4.1. Two-Dimensional Solution

In two dimensions, the direction u=(cosφ,sinφ) of a rod in the solution depends only on the angle φ between u and the *x*-axis. Applying the simple shear deformation

(17) $$\Lambda =\left(\begin{array}{cc} 1 & 0\\ \gamma & 1 \end{array}\right)$$

of strain *γ*, the particle orientation becomes $u_{\gamma }=\left(\cos\varphi_{\gamma },\sin\varphi_{\gamma }\right)$ with

(18) $$\varphi_{\gamma }=\arctan\left(\frac{\gamma \cos\varphi +\sin\varphi }{\cos\varphi }\right)\;$$

If we assume the system to be isotropic before the deformation, $f_{0}\left(\varphi \right)=1/\left(2\pi \right)$, we obtain a shear-induced dependence

(19) $$f_{\gamma }\left(\varphi \right)=\frac{1}{2\pi }|\frac{d\varphi_{-\gamma }}{d\varphi }|=\frac{1}{2\pi }\frac{\sec^{2}\varphi }{1+{\left(\gamma -\tan\varphi \right)}^{2}}$$

of the angular distribution function on φ. As *γ* increases, $f_{\gamma }\left(\varphi \right)$ turns into a double-peaked distribution for the ordered system, as illustrated in Figure 1b. The nematic order parameter $S\left(\gamma \right)=2\lambda_{1}-1$, associated to the distribution $f_{\gamma }$, is determined by the largest eigenvalue $\lambda_{1}$ of the second-rank ordering tensor

(20) $$\int \;\;\;d\varphi \;f_{\gamma }\left(\varphi \right)uu=\frac{1}{4+\gamma^{2}}\left(\begin{array}{cc} 2 & \gamma \\ \gamma & 2+\gamma^{2} \end{array}\right)\;,$$

which yields

(21) $$S\left(\gamma \right)=\frac{\gamma }{\sqrt{4+\gamma^{2}}}\;,$$

shown in Figure 1a. The corresponding eigenvector gives the director

(22) $$d\left(\gamma \right)\propto \left(\begin{array}{c} \sqrt{4+\gamma^{2}}-\gamma \\ 2 \end{array}\right)$$

(or nematic axis) of the nematic solution.

#### 4.4.2. Three-Dimensional Solution

Simple shear of the form

(23) $$\Lambda =\left(\begin{array}{ccc} 1 & 0 & 0\\ 0 & 1 & 0\\ \gamma & 0 & 1 \end{array}\right)$$

leaves the azimuthal angle $\varphi_{\gamma }=\varphi$ at its initial value and changes the polar angle as

(24) $$\theta_{\gamma }=\arccos\left(\frac{\cos\theta +\gamma \cos\varphi \sin\theta }{\sqrt{\sin^{2}\theta +{\left(\cos\theta +\gamma \cos\varphi \sin\theta \right)}^{2}}}\right)\;.$$

This leads to the strain-dependent angular distribution

(25) $$\begin{array}{cc} f_{\gamma }\left(\varphi ,\theta \right)\sin\theta & =\frac{1}{4\pi }|\frac{\partial (\varphi_{-\gamma },\theta_{-\gamma })}{\partial (\varphi ,\theta )}|\sin\theta_{-\gamma }\\ & =\frac{2\sin\theta }{\pi }{\left[4+\gamma^{2}-\gamma^{2}\cos\left(2\theta \right)-2\gamma^{2}\cos\left(2\varphi \right)\sin^{2}\theta +4\gamma \cos\varphi \sin^{2}\left(2\theta \right)\right]}^{-3/2} \end{array}$$

of an initially isotropic three-dimensional solution, $f_{0}=1/\left(4\pi \right)$. Note that $f_{\gamma }\left(u\right)$ is not axially symmetric with respect to the director d, but is characterized by biaxial order, which is typically quantified in terms of the second-rank ordering tensor. For our purpose, however, it suffices to estimate the degree of shear-induced alignment by the scalar order parameter *S* as follows. For given strain *γ*, it exhibits a maximum at $\left\{\varphi_{\gamma }^{*},\theta_{\gamma }^{*}\right\}$ with $\varphi_{\gamma }^{*}=0$ and

(26) $$\theta_{\gamma }^{*}=\arctan\left(\frac{1}{2}\sqrt{4+\gamma^{2}}-\frac{\gamma }{2}\right)\;,$$

thus determining to the director $d\left(\gamma \right)=\left(\sin\theta_{\gamma }^{*},0,\cos\theta_{\gamma }^{*}\right)$, which exactly corresponds to Equation (22) for the director of the sheared two-dimensional solution. As the distribution $f_{\gamma }\left(\varphi ,\theta \right)$ is not rotationally symmetric with respect to d(γ), an analytical expression for S(γ) cannot be derived. For small deformations, however, one obtains

(27) $$\int \;\;d\varphi \;f_{\gamma }\left(\varphi \right)uu∼\frac{1}{3}𝟙+\frac{\gamma }{5}\left(e_{1}e_{3}+e_{3}e_{1}\right)$$

to linear order in *γ*, where $e_{1}$ and $e_{3}$ denote the unit vectors parallel to the *x* and *z* axis, respectively. From its largest eigenvalue $\lambda_{1}=1/3+\gamma /5$ we obtain the linear scaling relation for S(γ)=(1/2)(3λ-1), given in Equation (3). In Figure 1, this asymptotic relation is compared with the numerically obtained S(γ), which reveals that it provides a good approximation up to γ≈1.

### 4.5. Unit-Cell Approach

**Figure 6:**
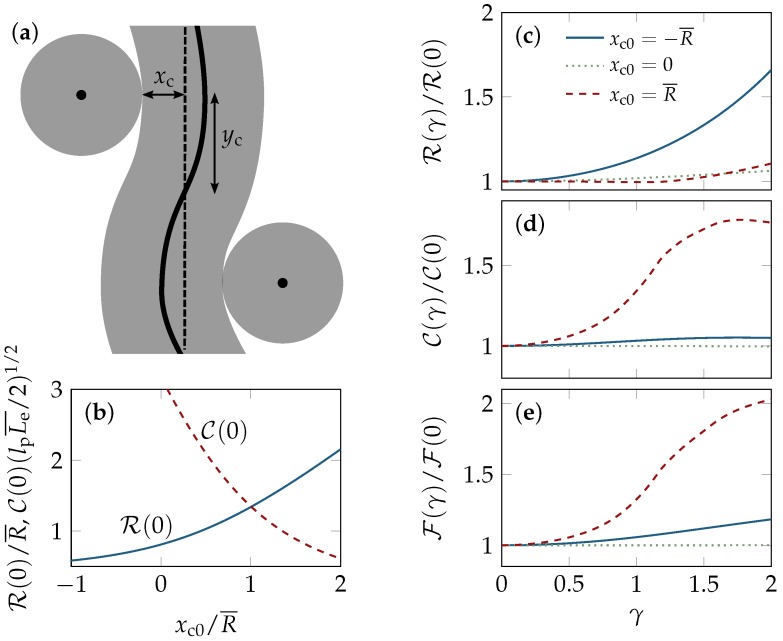
Unit-cell model by Fernández et al. [21]. (**a**) The test tube is deformed by two confining tubes (Sketch adapted from Reference [21]); (**b**) The zero-strain values of the average tube radius $\bar{R}\left(\gamma =0\right)$ and the mean curvature $\bar{C}\left(\gamma =0\right)$ varies with the unit-cell conformation, which is characterized by the deflection $x_{c0}$. Shearing of the network is mimicked by an affine displacement of the contact points with (or centers of) the confining tubes. There is a trade-off between bending and confinement, since a more strongly bent conformation allows for a wider tube, which, on average, gives rise to a strain-induced tube dilation and bending, quantified by (**c**) the average tube radius $\bar{R}\left(\gamma \right)$ and (**d**) the mean curvature $\bar{C}\left(\gamma \right)$ with strain *γ*; (**e**) The total free energy F(γ) increases upon shearing as required by mechanical stability. All curves were computed numerically from the full non-linear theory.

The non-affine tube deformation caused by a macroscopically imposed simple shear deformation is estimated using the unit-cell approach by Fernández et al. [21]. Namely, we minimize the free energy of a test tube clamped between two neighbor tubes that change their positions affinely with the applied strain *γ*, thereby exerting a force on the test tube, which bends accordingly. To treat large polymer deformations exactly, the polymer segment is modeled as an Euler-Bernoulli beam. Then, the tube backbone contour is obtained for strains *γ* up to ≈2 by solving the equation of elastica with forces acting on the collision points between the test tube and its confining neighbors. Assuming that the deformation happens in a quasi-static fashion, lateral friction is neglected and the test polymer remains equilibrated in its tube. The value of the constraining forces is determined by minimizing the test tube’s free energy comprising bending and confinement contributions. Following Reference [21], Figure 6 illustrates the “test tube” of the unit-cell model clamped between two neighboring tubes. Affine shearing of the polymer solution translates to an affine displacement of the confining tubes (whether the centers or contact points with the test tube are displaced does not matter much). Due to the mutual balance of enthalpic (backbone bending) and entropic (tube size) contributions to the free energy, shearing induces a strongly non-affine local polymer deformation, in this model. Averaging over a representative set of shear geometries, the deformation results in a dilation of the tube upon shear, which gives rise to a nonlinear softening at large strains, for a broad range of polymer concentrations and initial (equilibrium) tube conformations.

For moderate shear deformations, the model equations can be linearized and solved analytically. Starting point is the deflection $x_{c}+R$ of the tube backbone due to the force *f* between the colliding tubes, as given by Equation (9a) of Reference [21]. For small $y_{c}^{2}f/L_{p}$, where $2y_{c}$ is the distance between the tube–tube collisions along the backbone (see the sketch in Figure 6), the deflection becomes $x_{c}+R∼y_{c}^{3}f/\left(3L_{p}\right)$, which serves as a relation for the modified tube radius R. Note that positive deflections $x_{c}>0$ correspond to hairpin conformations. This yields the total free energy as the sum

(28) $$F∼y_{c}^{3}f^{2}/\left(6L_{p}\right)+L_{p}^{-1/3}y_{c}{\left[\left(2/3\right)y_{c}^{3}f/L_{p}-2x_{c}\right]}^{-2/3}$$

of the linearized bending free energy, i.e., expanded to lowest order in *f*, and the confinement free energy. Within the linearized theory, the lateral deflection $x_{c}+R∼y_{c}^{3}f/\left(3L_{p}\right)$ is varied to minimize F, so that the tube-deforming force *f* is determined by the equilibrium condition $\partial_{f}F=0$. If the backbone is only weakly bent, $y_{c}^{3}f/\left(L_{p}|x_{c}|\right)\ll 1$, this condition can be expanded to

(29) $$\frac{10y_{c}^{3}f}{9L_{p}|x_{c}|}∼-1+\sqrt{1+\left(10/27\right){\left(4\bar{R}/|x_{c}|\right)}^{8/3}}\;.$$

Inserted into the expression for the tube radius, we obtain its strain dependence $R\left(\gamma \right)/R\left(0\right)∼1+b_{R}\gamma^{2}$, Equation (5), with the zero-strain value

(30) $$\bar{R}\left(\gamma =0\right)/|x_{c0}|∼\frac{7}{10}+\frac{3}{10}\sqrt{1+\left(10/27\right){\left(4\bar{R}/|x_{c0}|\right)}^{8/3}}\;,$$

where $x_{c0}$ and $y_{c0}$ denote the collision coordinates for the unsheared solution. Following Reference [21], we here set $y_{c0}=\bar{L_{e}}/2$ and used that $\bar{L_{e}}=4L_{p}^{1/3}{\bar{R}}^{2/3}$, according to Equation (10). Besides the width of the tube, we characterize its conformation in terms of the mean curvature $\bar{C}$, defined through the average

(31) $${\bar{C}}^{2}\equiv \frac{1}{2y_{c}}\int_{0}^{y_{c}}\;\;\;\;ds\;{\left[r^{\prime \prime }\left(s\right)\right]}^{2}$$

along the backbone contour r(s) of the tube segment. Note that $y_{c}L_{p}{\bar{C}}^{2}$ is nothing but the bending energy of the tube segment (in natural units) as it appears in the unit-cell model. With the weak-force scaling of Equation (29), a similar calculation as for $\bar{R}\left(\gamma \right)$ yields the asymptotic scaling $\bar{C}\left(\gamma \right)/C\left(0\right)∼1+b_{C}\gamma^{2}$, Equation (6), with

(32) $$\bar{C}\left(\gamma =0\right)∼\frac{9|x_{c0}|/\bar{R}}{20\sqrt{6L_{p}\bar{L_{e}}}}\left[-1+\sqrt{1+\left(10/27\right){\left(4\bar{R}/|x_{c0}|\right)}^{8/3}}\right]\;.$$

Comparing the asymptotic scaling relations with the numerically solved full model, we find the quadratic strain dependencies in Equations (5) and (6) to be in good agreement with the full model. However, as the above weak-force criterion does not hold for typical model parameters, the zero-strain values $\bar{R}\left(\gamma =0\right)$ and $\bar{C}\left(\gamma =0\right)$ and the coefficients $b_{R}$ and $b_{C}$ obtained from the full model can deviate substantially from their asymptotic estimates given here. Their dependence on the ratio $x_{c0}/\bar{R}$ between the unperturbed tube deflection and the width of a straight equilibrium tube is shown in Figure 6. With $x_{c0}=-\bar{R}$, for instance, we have $y_{c}^{3}f/\left(L_{p}|x_{c0}|\right)\approx 2$ and, consequently, the asymptotic solutions $\bar{R}\left(\gamma =0\right)∼1.9\bar{R}$ and $\bar{C}\left(\gamma =0\right)∼0.39{\left(L_{p}{\bar{L}}_{e}/2\right)}^{-1/2}$ of the linear model markedly differ from the numerically obtained predictions $\bar{R}\left(\gamma =0\right)\approx 1.3\bar{R}$ and $\bar{C}\left(\gamma =0\right)\approx 1.4{\left(L_{p}{\bar{L}}_{e}/2\right)}^{-1/2}$ of the full model, see Figure 6b. Notably, $b_{C}$ can actually become negative as illustrated in Figure 6d.

We note that we quantify the geometry of the straight tube by means of the BCA predictions for $\bar{R}$ and $\bar{L_{e}}$, given in Equation (11), whereas the entanglement length in Reference [21] was defined as $\bar{L_{e}}={\left(L_{p}/\bar{\phi }\right)}^{1/4}$, which differs from Equation (10) by the numerical prefactor $2^{3/2}$. The slightly larger values for $\bar{L_{e}}$ and $y_{c0}$ used here have consequences for both the geometry and stability of the ground state of the unit cell, as described so far, which predicts an instability against shearing when the aspect ratio $|x_{c0}|/y_{c0}$, or, equivalently, the ratio $|x_{c0}|/\bar{R}$, exceeds a certain threshold value. In this regime the total model free energy decreases upon straining, as the test tube is widened to an unrealistically large volume that violates the constraint set by the imposed polymer concentration. As shown in Reference [21], this artifact of the simplified unit-cell geometry can be cured by accounting for the so-called “Doi–Kuzuu effect” [55], which relates the number of contacts between the tubes to the applied deformation. Upon large deformations, the test tube makes new lateral contacts with previously spatially separated tubes, which limits the lateral expansion to physically reasonable bounds and can effectively be accounted for by a renormalization of the unit cell parameters as a function of the strain. With this important amendment, the ground state is always stable and robust to moderate parameter changes. The resulting strain-dependent tube radius, curvature, and (clearly stable) total free energy are depicted in Figure 6, using typical values $L_{p}=17\;\mu m$ and $\rho =20\;\mu m^{-2}$ for the persistence length and the polymer length concentration, respectively, in Equation (11). The latter correspond to semi-dilute F-actin solutions of concentration c=0.5mg/mL, based on the estimate $\rho /c\approx 40\;\mu m^{-2}/\left(\mathrm{mg}/\mathrm{mL}\right)$ obtained from the molecular structure of the actin filaments [43].

It is worth noting that the Doi–Kuzuu effect affects so-called hairpin configurations (thermodynamically suppressed strongly contorted unit cell configurations [20,21]) differently from the typical configurations. We keep track of this by the factor $-x_{c}/|x_{c}|$ in the second term of the differential equation

(33) $$y_{c}^{\prime }\left(\gamma \right)=y_{{}_{c}0}\lambda_{y}^{\prime }\left(\gamma \right)-4\left(x_{c}/|x_{c}|\right)\left(y_{c}^{2}R/\xi^{2}\right)\lambda_{x}^{\prime }\left(\gamma \right)-4\left(y_{c}^{2}/\xi^{2}\right)R^{\prime }\left(\gamma \right)$$

that determines the longitudinal deformation of the unit cell (Equation (18) of Reference [21]). The first term on the right hand side represents the affine stretch by a factor $\lambda_{y}\left(\gamma \right)$, the last term corrects for the volume change of the unit-cell due to tube dilation/contraction. The second term accounts for the transversely approaching/distancing of the tube segments that follow the stretch factor $\lambda_{x}\left(\gamma \right)$, which effectively decreases/increases the distance $2y_{c}$ between the collision points. For a hairpin configuration, characterized by $x_{c}>0$, neighboring tubes are pushed aside, corresponding to an increasing $y_{c}$. It should also be noted that, apart from this topological distinction, the correction is of mean-field type. This restriction could tend to iron out a physically meaningful heterogeneous response of the polymer (tube) network. Beyond the mean-field approximation, unstable unit-cells might still exist locally and give rise to spontaneous network heterogeneities (without invoking any enthalpic attractions or crosslinkers). In a stationary shear flow, these might play an important role for the nucleation of shear bands, a phenomenon that is known to occur in densely entangled solutions of flexible polymers [56] and that was recently observed for entangled F-actin solutions [16]. It would be interesting to see, whether such effects could be grasped with the theory by pushing the treatment of the Doi–Kuzuu effect beyond the mean-field approximation.

Finally, we estimate the non-affine contributions to the nematic order parameter *S*, within the unit-cell approach. Unfortunately, finding the exact deformation field from the complex response of the tube backbone is not a straightforward task. To simplify the computation, we therefore follow Reference [21] and consider only a discrete set of test-tube segments with orientations along the three main stretch directions or principal axes of the simple shear deformation. Under the assumption that the average tube deformation can be described by a deformation tensor $\Lambda_{\gamma }=\Lambda_{\gamma }^{a}+\Lambda_{\gamma }^{n}$ that we decompose into its affine and non-affine contribution, respectively, we can approximately reconstruct the non-affine contribution $\Lambda_{\gamma }^{n}$ from the three principle shear transformations. We first relate these transformations for the stretching and compression along the principal axes of the affine deformation with the lab-frame coordinates. We denote these principal directions (i.e., the normalized eigenvectors of the affine Cauchy deformation tensor ${\left(\Lambda_{\gamma }^{a}\right)}^{T}\Lambda_{\gamma }^{a}$) by $v_{i}$, and the corresponding stretch factors (the eigenvalues) by $\lambda_{i}$. The index convention is that i=1,2,3 represent the stretch, compression, and neutral axis, respectively. Note that $v_{i}$ gives the direction *before* the shear deformation and yields the orientation $w_{i}\equiv \lambda_{i}^{-1}\Lambda_{\gamma }^{a}v_{i}$ after an affine deformation $\Lambda_{\gamma }^{a}$ has been applied. For a given point $r_{0}$ in the lab frame, the model now takes its principal-axes coordinates $v_{i}\;\cdot \;r_{0}$ before the deformation and computes its principal-axes coordinates $w_{i}\;\cdot \;r_{\gamma }$ at strain *γ*. With the above introduced decomposition into an affine and a non-affine contribution to the deformation tensor, $r_{\gamma }=\left(\Lambda_{\gamma }^{a}+\Lambda_{\gamma }^{n}\right)\;\cdot \;r_{0}$, we obtain

(34) $$w_{i}\;\cdot \;r_{\gamma }=\lambda_{i}v_{i}\;\cdot \;r_{0}+\sum_{j}w_{i}\;\cdot \;\left(\Lambda_{\gamma }^{n}\;\cdot \;v_{j}v_{j}\;\cdot \;r_{0}\right)\;.$$

The unit operator $𝟙=\sum_{j}v_{j}v_{j}$ was inserted to reintroduce the coordinates $v_{j}\;\cdot \;r_{0}$. This equation must be solved for the nine components of $\Lambda_{\gamma }^{n}$, which is indeed possible if we insert the end-to-end vector of the tube segments for $r_{0}$ and $r_{\gamma }$ and exploit the available information for all three principal axes (i=1,2,3). Here, another technical detail of the unit-cell model has to be taken into account: fixing the (unperturbed) tube direction to $v_{i}$ yields two possible unit-cell conformations, depending on the orientation of the trihedron built by the three principal axes. We average over these two geometries to obtain the mean change of the tube segment orientation that we can use in Equation (34). With the full deformation tensor $\Lambda_{\gamma }=\Lambda_{\gamma }^{a}+\Lambda_{\gamma }^{n}$ at hand, the non-affine strain dependence S(γ) of the nematic order parameter is readily computed by applying the same procedure as in Section 4.4 for the purely affine deformation. Combining S(γ) and $\bar{R}\left(\gamma \right)$ predicted by the unit-cell approach, we eventually obtain the order-dependent mean tube radius $\bar{R}\left(S\right)$ that is shown in Figure 1.

## Abbreviations

The following abbreviations are used in this manuscript:

WLC: Wormlike chain
BCA: Binary collision approximation
MC/BD: Monte Carlo/Brownian dynamics
